# Adjuvant Probiotics and the Intestinal Microbiome: Enhancing Vaccines and Immunotherapy Outcomes

**DOI:** 10.3390/vaccines5040050

**Published:** 2017-12-11

**Authors:** Luis Vitetta, Emma Tali Saltzman, Michael Thomsen, Tessa Nikov, Sean Hall

**Affiliations:** 1 Sydney Medical School, The University of Sydney, Sydney 2006, Australia; esal4025@uni.sydney.edu.au (E.T.S.); mtho0952@uni.sydney.edu.au (M.T.); 2 Medlab Clinical Ltd., Sydney 2015, Australia; tessnikov@gmail.com (T.N.); sean_hall@medlab.co (S.H.)

**Keywords:** vaccines, probiotics, infections, immunological tolerance

## Abstract

Immune defence against pathogenic agents comprises the basic premise for the administration of vaccines. Vaccinations have hence prevented millions of infectious illnesses, hospitalizations and mortality. Acquired immunity comprises antibody and cell mediated responses and is characterized by its specificity and memory. Along a similar congruent yet diverse mode of disease prevention, the human host has negotiated from in utero and at birth with the intestinal commensal bacterial cohort to maintain local homeostasis in order to achieve immunological tolerance in the new born. The advent of the Human Microbiome Project has redefined an appreciation of the interactions between the host and bacteria in the intestines from one of a collection of toxic waste to one of a symbiotic existence. Probiotics comprise bacterial genera thought to provide a health benefit to the host. The intestinal microbiota has profound effects on local and extra-intestinal end organ physiology. As such, we further posit that the adjuvant administration of dedicated probiotic formulations can encourage the intestinal commensal cohort to beneficially participate in the intestinal microbiome-intestinal epithelia-innate-cell mediated immunity axes and cell mediated cellular immunity with vaccines aimed at preventing infectious diseases whilst conserving immunological tolerance. The strength of evidence for the positive effect of probiotic administration on acquired immune responses has come from various studies with viral and bacterial vaccines. We posit that the introduction early of probiotics may provide significant beneficial immune outcomes in neonates prior to commencing a vaccination schedule or in elderly adults prior to the administration of vaccinations against influenza viruses.

## 1. Introduction

The history of vaccines is reported to date back to the late 18th century [1]. The contentious issues relevant to the value of vaccines that have been advanced at different times, notwithstanding the administration of vaccinations have comprised one of the brightest episodes in medicine’s contribution to human health and longevity. Although they are one of the most beneficial and cost-effective disease prevention methods to date, there is still the capacity to optimise the protective effect of immunizations in countries with already maximal levels of childhood immunization [2,3]. As such, modalities, which increase the efficacy of immunizations will help protect the substantial proportion of children who remain vulnerable and susceptible to infections [4].

Vaccine-induced immune effectors are essentially antibodies that are produced by B lymphocytes and which proficiently and specifically bind to a toxin or a pathogen [5]. Additional potential effectors include cytotoxic CD8^+^ T lymphocytes that may limit the spread of infectious agents by identifying and neutralizing infected cells or by secreting specific antiviral cytokines. The generation and maintenance of both B and CD8^+^ T cell responses is supported by growth factors and signals provided by CD4^+^ T helper lymphocytes. CD4^+^ T helper lymphocytes commonly subdivide into T helper 1 and T helper 2 subtypes. These effectors are controlled by regulatory T cells (Treg) that are involved in maintaining immune tolerance [6]. Most antigens and administered vaccines trigger both B and T cell responses. As such there is no rationale for vaccines to oppose antibody production via humoral immunity reactions and T cell responses via *cellular immunity*. Furthermore CD4^+^ T cells activation are compulsory for most antibody responses, while antibodies exert significant influences on T cell responses to intracellular pathogens [7].

The intestines of humans is the anatomical site for the most complex and extensive collection of microscopic entities, that includes, bacteria, archaea, microbial eukaryotes and viruses, collectively termed as the microbiome [8]. It has been reported that the intestinal microbiome of healthy individuals is dominated by bacterial species from the *Bacteriodetes* and *Firmicutes* phyla, with representation from additional less dominant phyla, namely *Actinobacteria*, *Fusobacteria*, *Proteobacteria* and *Verrucomicrobia* [9]. In the gut there is an established complex and refined immune system that protects from pathogen infections, while maintaining tolerance to food, environmental and non-pathogenic bacterial antigens. The mucus layer over the intestinal epithelia contains antimicrobial effectors and secretory IgA and is the first defensive component of the intestines [10]. The intestinal epithelia through its secretory antibacterial peptides and innate and adaptive immune system network of cells regulate intestinal immunity. Intestinal mucosal immune cells are specifically organized to form the gut-associated lymphoid tissue (GALT), where immune cells are activated by bacterial antigen triggers. Probiotics are reported to improve intestinal microbial profiles by balancing and promoting homeostasis in the microbial community following perturbing events that can trigger intestinal microbial dysbiosis, as well as intestinal epithelial cell dysbiosis (a gut barrier abnormality); such as the administration of antibiotic therapy [11]. Probiotic administration is posited to positively influence local immunological equilibrium and local and extra-intestinal physiology [12].

Probiotics can be viewed as biological response modifiers [13,14] that are capable of exerting their health effects via a combination of mechanisms. These mechanisms include competitive displacement of pathogens in the intestinal lumen, epithelia and intestinal mucosa; the assembly of antimicrobial proteins toxic to pathobionts (i.e., bacteria that are capable of pathogenic activity against the host), the production of metabolic substrates for the maintenance of epithelial barrier and mucosal integrity and the modulation of immune function. Altering the intestinal microbial composition to exert positive health benefits to the host, the exact mechanism by which probiotics affect their influence, remains incompletely understood. However, clinical and experimental data indicates an immunomodulatory effect on the host immune system is involved [4]. The interactions between the human host and the intestinal microbiome for example, afford the human host the necessary cues for the development of regulated signals that in part are induced by reactive oxygen species. This regulated activity then promotes immunological tolerance and metabolic regulation and stability, which helps to establish control of local and extra-intestinal end-organ (e.g., liver, kidney and mucosal immunity) physiology. Therefore, pharmacobiotics, defined as the targeted administration of live probiotic cultures, is an advancing area of potential therapeutics, either directly or as adjuvants [13].

**Figure 1:**
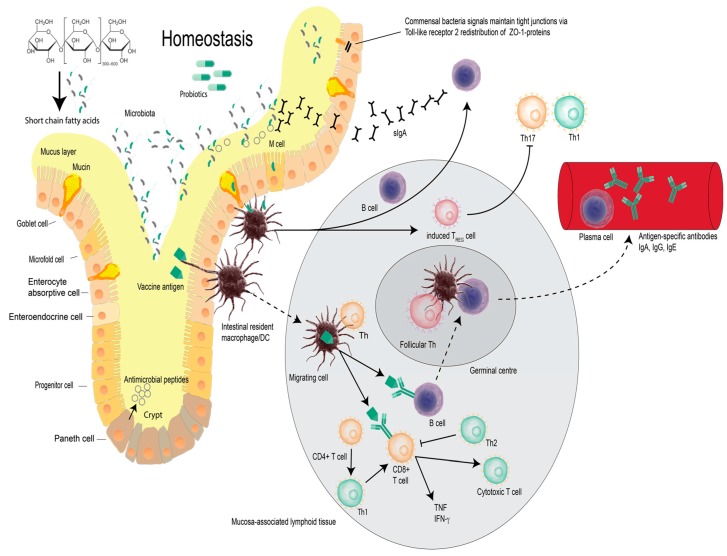
Vaccine, epithelial barrier function and intestinal homeostasis. Intestinal immunological homeostasis is maintained by a complex interplay of the intestinal epithelial and localised immune cells. Antigen presenting macrophages/dendritic cells in the mucosa and associated lymphoid tissue, Peyer’s patches, mesenteric lymph nodes and lymphoid follicles co-ordinate immunological responses to maintain local homeostasis. Intestinal homeostasis supports the vaccine-induced production of antigen-specific antibodies via the presentation of vaccine antigen by migrating dendritic cells to B cells in the mucosa associated lymphoid tissue. Vaccine antigens also stimulate naïve CD4^+^ and CD8^+^ T cells to differentiate into cytotoxic T cells and to release antimicrobial cytokines. This figure was constructed and adapted from relevant published works [18,19,20].

The scientific literature teaches that the administration of probiotics can modulate both innate and adaptive immunity (Figure 1). Innate immunity is defined as the part of the immune system that is non-specific and not dependent on prior sensitization to an antigen. Whereas adaptive immunity provides protection from an infectious disease agent, with responses that are mediated by B- and T- lymphocytes following exposure to specific antigen(s), and is importantly characterized by immunological memory [15]. Klein et al., has previously demonstrated that when young adults were administered a daily probiotic supplement compared to placebo, a significantly elevated proportion of granulocytes and monocytes showed phagocytic activity [16]. These observations were affirmed by the findings of Gill et al., who reported a significant increase in serum antibody responses to antigens (administered orally and systemically) in mice treated with probiotics [17].

## 2. Vaccines, Probiotic Bacteria and Modulation of Immunity

Vaccines in their current use are described as live attenuated, killed/inactivated, or subunit (recombinant) in kind and are mainly administered parenterally [21]. In Australia vaccines are predominantly administered intramuscularly. Only a few vaccines are given subcutaneously, either orally or intradermally [22]. Some vaccines have been reported to exhibit poor immunogenicity and a limited capacity to induce mucosal and cell mediated immunity effects. It is difficult to group vaccines that exhibit poor immunogenicity because immune responses to vaccines can be affected by numerous factors [23]. For example early life immune responses to vaccines have been reported to be characterized by age dependent limitations of the magnitude of the response. That is antibody responses to most capsular polysaccharide antigens are not elicited during the first two years of life which most likely reflects slow maturation of the splenic marginal zone, limited expression of CD21 on B cells and limited expression of the complement factors [24]. Even the implementation of the most potent glyconjugate vaccines elicit markedly lower IgG responses in infants [25]. Early life antibody responses are very much directly influenced by prenatal/gestational age and post-natal age of immunization. In late adulthood, changes in vaccine responses are age associated. Innate and cell mediated cellular immunity responses decline with age, which increases the frequency and severity of infections and reduces the protective effects of vaccinations. The aging process affects the magnitude and the persistence of antibody responses to protein vaccines [26], tetanus and tick borne encephalitis vaccines [27] and pneumococcal capsular polysaccharide vaccines [28].

Enhancing mucosal immunity offers an effective strategy for preventing pathogen adhesion to host tissues (i.e., colonization) and the maximization of vaccine efficacy against infections, which rely on the adhesion of pathogens such as the invasive disease caused by the pneumococcus pathobiont [29]. It has been demonstrated that probiotic bacteria can influence immune function by direct and indirect actions [30]. The direct effects include encouraging the intestinal commensal microbiota to alter the profile of pathogen associated molecular patterns (PAMPs) presented to the gut associated lymphoid tissue (GALT). Indirect effects may arise from microbial products such as short chain fatty acids (SCFAs) [31]. Evidence from animal models (with germ free mice) that are highly susceptible to numerous viral infections, including influenza, indicates that the resident intestinal microbiota shapes anti-viral defences and modulates the outcome of viral infections [32]. Experiments conducted with specific pathogen-free mice that were treated with antibiotics support these observations. Antibiotic treated specific pathogen-free mice given a sub-lethal dose of PR8 virus (influenza virus variant) had impaired generation of virus specific antibodies, cluster of differentiation CD4^+^T and CD8^+^T cell responses and delayed viral clearance [32]. A further study reported that pathogen-free mice treated with antibiotics showed reduced migration of respiratory dendritic cells from the lungs to the draining lymph node during influenza infection [33]. Thus, as a result, a reduction in the priming of naïve antigen specific CD8^+^T cells was observed.

Further studies with laboratory animals administered antibiotics have identified specific classes of bacteria that are involved in maintaining immunity against viral infections. A report demonstrated that intestinal bacteria sensitive to neomycin completely eliminated *Lactobacillus* spp. and resulted in the impairment of influenza-specific CD8^+^ T cell responses. This result suggested that neomycin sensitive bacteria in the gut supports the immune response to an influenza infection [32]. Intestinal microbes have also been suggested to support immune responses against viral infections through effects on inflammasome-mediated cytokine production and release. In studies where antibiotic-treated mice showed reduced levels of interleukin-1b secretion in the lung during infection with the influenza virus, it is suggested that resident intestinal bacteria support cytokine production and release [29]. Furthermore, it was noted that intestinal bacteria can release low-levels of pattern recognition receptor (PRR) ligands that then provide signals for inflammasome-mediated cytokine release (as an exemplar, in the lung during infections with influenza virus). Reports show that the activity of respiratory dendritic cells is regulated during activation of adaptive immunity against the virus [32]. Resident intestinal bacteria that participate in shaping immune defences form the basis of the hypothesis that probiotic bacteria may modulate responses to infections or vaccinations. Interestingly though, the mechanisms by which probiotics modulate the immune system, particularly in the context of vaccination, remain to be explicitly clarified.

A recent animal study demonstrated that the probiotic, *Lactobacillus gasseri* was able to trigger the diversification of B cell populations in the lamina propria of the murine colon in vivo. In addition, *L. gasseri* was also proposed as a vaccine vector for oral immunization against mucosal pathogens [34]. In a further similar study it was demonstrated that *Lactobacillus paracasei* subsp. *paracasei* NTU 101 that had been administered daily to mice for three to nine weeks induced a more pronounced effect between CD4^+^ T cells and dendritic cells and enhanced the proliferation of CD4^+^T cells and B cells [35].

Probiotics have been demonstrated to have pleiotropic effects on innate and adaptive immune responses in vitro and in vivo [36]. Probiotics can mediate immunological effects directly through their interaction with intestinal immune cells and epithelial cells or indirectly through modulation of the intestinal microbiome [37,38]. Additionally, the action of probiotics in maintaining health has been posited to be the result of a combination of multiple site effects, namely on the intestinal microbiome, the integrity of the epithelial barrier and immune tissue modulation. Interactions between the intestinal microbiota and the immune system are recognised as vitally important for the development of healthy immune responses and also the maintenance of immune tolerance. Studies have repeatedly shown that intestinal barrier dysbiosis can lead to chronic inflammatory conditions such as allergic disease and inflammatory bowel disease (e.g., Crohn’s disease, ulcerative colitis), most probably as a result of unbalanced immune activity and regulation [4,39,40].

**Figure 2:**
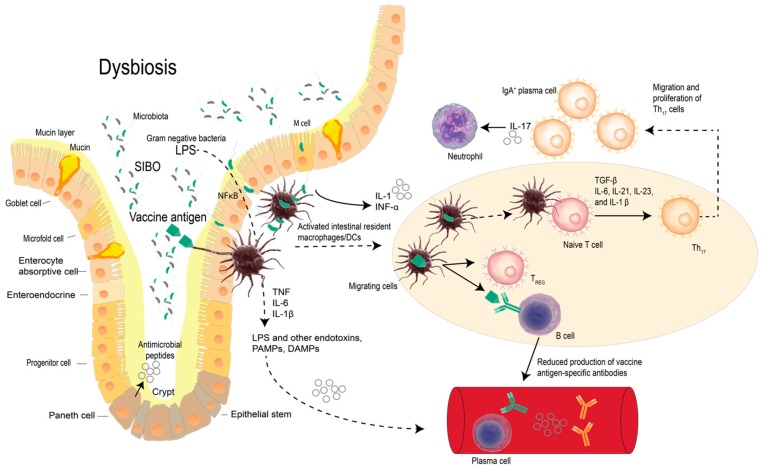
Intestinal microbiome dysbiosis compromises the effectiveness of vaccine antigens secondary to systemic consequences of a chronic inflammation of the intestinal tract. In chronic intestinal inflammatory states, Tregs can suppress immune responses against antigens and this may limit the efficacy of vaccines when vulnerability signals are not sufficient to elicit vaccine-induced immunity via production of vaccine antigen-specific antibodies. A plausible posit is that probiotics can improve vaccine responses by encouraging the intestinal microbiome to restore eubiosis that restores intestinal immunological homeostasis. This figure was constructed and adapted from relevant published works [18,19,20,45,46,47,48].

The most extensively investigated probiotic bacteria in animal models and clinical trials are those from the *Lactobacilli* and *Bifidobacteria* species. The immune-modulatory potential of probiotic bacteria very much highlights the beneficial effects that some probiotics provide in the prevention of allergic disease [41]. The intestinal bacteria (whether pathogenic or commensal) interact with the intestinal mucosal lymphoid system through PRRs that are expressed on specialized intestinal epithelial M cells and dendritic cells. Antigen presenting cells in return direct host immune responses [41,42]. The signalling events are central to the maintenance of inter-intestinal and extra-intestinal immune homeostasis, [43] allowing host protection against intestinal pathogens while at the same time preventing undesirable immune activations through the induction of tolerogenic responses. Relevant to vaccine development, the role of the human microbiome in inducing beneficial systemic and mucosal immune responses has become increasingly evident, with probiotics posited to have a potential role as novel vaccine adjuvants [44] (Figure 2).

## 3. Probiotics and Vaccines 

### 3.1. Probiotics and Vaccines in Infants

Studies report that different probiotic strains show adjuvant efficacy for different types of vaccines (Table 1). Lactobacillus rhamnosus strain GG administered to 2–5-month-old infants immediately before receiving the oral rotavirus vaccine and for the subsequent 5 days were reported to have significantly increased rotavirus-specific immunoglobulin M antibody secreting cells 8 days after vaccination compared to placebo [49]. Furthermore, the study also reported that Lactobacillus rhamnosus GG administration was associated with a trend for higher rotavirus-specific IgA antibody titres. In contrast, there was no effect (i.e., viral-specific IgA antibodies) observed in 2–5-year-old infants that were administered Bifidobacterium breve strain BBG-01 for 4-weeks in response to oral cholera vaccine [50].

Other studies with infants parenterally administered vaccines (Table 1) have demonstrated contentious adjuvant efficacy. One study investigated the administration of a combination of probiotics and a prebiotic (galacto-oligosaccharide) [56] on antibody responses to diphtheria/tetanus/haemophilus influenza type b vaccination in allergy prone new-born infants. The pregnant mothers received the formulation (without the prebiotic) in the last month of their pregnancy and the newborn infants received the full formulation for their first six months. Vaccines were administered at three, four and five months of age and antibody titers were measured at 6 months. A protective Hib-specific IgG antibody response (>1 μg/mL) occurred more frequently in the probiotic group over placebo. However, there were no vaccine-specific antibody titers observed between the groups. In another study, Lactobacillus acidophilus was administered to allergy-prone infants for the first 6 months of life and the response to the tetanus vaccine was assessed at two, four and six months of age [54]. The probiotic decreased the IL-10 response to tetanus toxoid antigen at 6-months and reduced IL-5 and transforming growth factor-b release by peripheral blood mononuclear cells following stimulation with Staphylococcal enterotoxin B when compared to placebo. In a further study [53], 4-month old infants were provided with a cereal containing Lactobacillus paracasei ssp. paracasei strain F19 vs. a placebo cereal only group for nine months. The study reported that in infants immunized with diphtheria/tetanus/toxoid/acellular pertussis/polio/haemophilus influenza type b at three, 5.5 and 12 months of age, no significant effect on antibody titers were observed. However, when the groups were adjusted for breastfeeding duration, the results suggested that the probiotic enhanced anti-diphtheria antibody titers in the infants that had been breastfed for less than 6 months. Moreover, a similar effect was observed for the tetanus antigen, but there was no such effect observed for the probiotic group on haemophilus influenza type b vaccination.

In response to hepatitis B vaccination in allergy-prone infants formula-fed supplemented with Bifidobacterium longum BL999 and Lactobacillus rhamnosus LPR or a control formula without probiotics in the first 6-months of life in new-borns, there was no reported significant trend for the probiotic formulation to increase hepatitis B virus surface antibody responses in the infants that received hepatitis B+ hexavalent diphtheria-tetanus-acellular pertussis combination vaccine [55]. There was no effect of probiotics in infants receiving the monovalent hepatitis B vaccine. In a study with a multi-strain probiotic formulation (Lactobacillus acidophilus ATCC4356/Bifidobacterium bifidum DSMZ20082/Bifidobacterium longum ATCC157078/Bifidobacterium infantis ATCC15697) [4] administered for 5 months starting at two months prior to vaccination against mumps/measles/rubella/varicella, there was a trend towards a greater percentage of infants reaching protective IgG antibody titers three-months post-vaccination in the probiotic group only, when all antibody results were combined.

From a study within a larger clinical trial, the Lactobacillus rhamnosus GG (LGG) effect on immune responses to tetanus, Haemophilus influenza type b and pneumococcal conjugate vaccines in infants was assessed, in conjunction with the impact of maternal LGG supplementation in preventing the development of atopic eczema in infants at high-risk for developing allergic disease [52]. The study reported that maternal LGG supplementation was associated with reduced antibody responses against tetanus, Haemophilus influenza b, and pneumococcal conjugate compared to placebo treatment but not total IgG levels. Further, maternal LGG supplementation was also associated with a trend of an increased number of tetanus toxoid-specific T regulatory cells in the peripheral blood compared to placebo-treated infants. Overall, the results suggest that maternal LGG supplementation may not be beneficial in terms of improving vaccine-specific immunity in infants.

A clinical study with infants investigating if probiotics could maintain their immune-stimulating effects, children were supplemented for at least four months with one of two products, a low-fat milk fermented by Streptococcus thermophilus (as control) and a low-fat milk fermented by *S. thermophilus* containing Lactobacillus casei, Lactobacillus acidophilus, oligofructose and inulin added after the fermentation process. The study concluded that supplementation with the standard fermented milk and probiotics was not of benefit. The naturally high rate of early microbial exposure in infants and children from a population of low socio-economic status that tend to live in a less hygienic environment may account for the absence of an additional immune-stimulating effect by supplementary probiotics.

Previous studies have suggested that infants raised in developing countries develop higher antibody titres following immunisations than infants from more developed regions [51]. Termed the hygiene hypothesis, IgG2 production is favoured following stimulation of Th1 immune responses as a result of repeated exposure to viral and bacterial infections and repeated antigen production [4]. A preference for the Th2 immune response profile is associated with improved public health and hygienic environments in developed countries and contributes to antibodies’ reduced ability to sufficiently respond to infectious or viral disease exposure [16,57,58]. These indications were further supported by a study completed by Pérez et al., in Argentina, demonstrating probiotic supplementation showed no effect on antibody responses following the DTP-Hib and 23-valent pneumococcal vaccine in children living in a region with a lower socio-economic status [51].

Overall there are trends from these studies (Table 1) towards better responses to vaccinations in infants administered probiotic formulations. However, limitations are evident and as such, further clinical studies are warranted. The literature is consistent in the non-occurrence of adverse effects reported in either treatment groups in infant studies, supporting the safety of probiotic supplementation in healthy infants [59].

### 3.2. Probiotics and Vaccines in Adults

A number of studies have also investigated the effect of probiotics in adults administered vaccines orally, parenterally and nasally (Table 2). Fermented milk containing Lactobacillus acidophilus La1 and Bifidobacterium breve Bb12 was consumed for three weeks and significantly increased the vaccine-specific serum IgA titers to an attenuated Salmonella typhi Ty21a oral vaccine given on days 7, 9 and 11 of 21 [60]. In a separate study, LGG taken for seven days increased vaccine-specific IgA antibodies to the Salmonella typhi Ty21a oral vaccine that was administered on days 1, 3 and 5 [61]. However, no effect was reported for *Lactococcus lactis* or LGG on the numbers of vaccine specific IgA, IgG and IgM antibody secreting cells 7-days post-vaccination [61].

Vaccine specific IgA titer to an oral poliovirus vaccine was increased by LGG and *L. casei* CRL431 during a five-week intervention with a live attenuated poliomyelitis virus vaccine administered on day 8 [63]. Subjects taking the probiotics had a significantly greater increase in neutralizing antibodies compared to placebo. In addition, a minor effect was recorded on poliovirus serotype-1-specific IgG and on serotype-2-and-3-specific IgM antibody titers [63].

Strain specific effects of probiotics in response to an oral cholera vaccine have been investigated by by Paineau and colleagues [65]. In a study with healthy volunteers allocated to one of seven probiotic strains (i.e., strains from the *Lactobacillus* and *Bifidobacterium* genera) or placebo for 3-weeks, subjects received the oral cholera vaccine on days 7 and 14. *B. lactis* Bi-04 and *L. acidophilus* La-14 significantly increased vaccine-specific serum IgG antibody levels at 3-weeks. Moreover, there was a similar trend observed for *B. lactis* Bi-07 and *L. plantarum* Lp-115. Probiotics therefore had no significant effects on vaccine-specific serum IgA or IgM antibodies [65].

A study in an elderly patient population [66] was conducted to investigate probiotic responses to influenza vaccinations. Eighty-six and 222 elderly volunteers consumed either a fermented dairy drink (containing the probiotic strain *Lactobacillus casei* DN-114 001) with yoghurt ferments or a control dairy product twice daily for a period of 7-weeks (pilot phasse) or 13-weeks (confirmatory phase), respectively. Vaccination occurred after four weeks of product consumption. In the pilot phase of the study, the influenza-specific antibody titers increased after vaccination and were consistently higher in the probiotic product group compared to the control group under product consumption. Likewise, in the confirmatory phase, titers against the B strain increased significantly more in the probiotic group than in the control group at three, six and nine weeks post-vaccination. Significant differences in sero-conversion between the groups by intended to treat analysis were still found five months after vaccination. Similar geometric mean titre results were observed for the H3N2 and H1N1 strains, very much confirming the results of the pilot study.

It is widely accepted that ageing is associated with immune dysregulation and leads to increased infection rates and reduced vaccination effectiveness. An early study investigated a nutritional supplement that contained probiotics on the immune response and cytokine production [62]. Elderly subjects were provided with a nutritional formula (in addition to their regular diet), which contained 31 g protein, 120 IU vitamin E, 3.8 μg vitamin B12, 400 μg folic acid, Lactobacillus paracasei (NCC 2461) and 6 g of fructo-oligosaccharides. The study reported an increase in NK activity in supplemented subjects and a decrease for non-supplemented individuals. IL-2 production by PBMC and the proportion of T cells with NK activity decreased in controls and did not change in supplemented subjects. Supplemented subjects reported less infective episodes than non-supplemented individuals.

An interventional study with *L. casei* Shirota with elderly nursing home residents was completed to reduce susceptibility to respiratory symptoms and improve their immune response to influenza vaccination [68]. Subjects were randomized to receive the probiotic or the placebo for six months. At three weeks all subjects were administered influenza vaccination. The results showed that daily consumption of a fermented milk drink that contains *L. casei* Shirota had no statistically or clinically significant effect on protection against respiratory symptoms.

An elderly cohort [74] was randomized to receive *L. paracasei* MCC1849 cells or a placebo jelly without probiotic for 6-weeks. Three weeks following the start of supplementation, all subjects received a trivalent influenza vaccination (A/H1N1, A/H2N3 and B). There were no significant differences in immune parameters, including antibody responses against the vaccination between the groups. In the subgroup of the oldest of the old, defined as ≥85 years of age, the antibody responses to the A/H1N1 and B antigens were improved only in the probiotic group. No significant effects of non-viable *L. paracasei* MCC1849 were observed in the elderly[74].

A study investigated the ability of a combination B. lactis (BB-12^®^) and *L. paracasei* ssp. paracasei (*L. casei* 431^®^) formula to modulate the immune system after vaccinations in healthy subjects [69]. Changes from baseline in vaccine-specific plasma IgG, IgG1 and IgG3 were observed to be significantly greater in both probiotic groups compared to placebo. Significantly greater mean fold increases for vaccine-specific secretory IgA in saliva was also observed in both probiotic groups. Similar results were observed for total antibody concentrations. No differences were found for plasma cytokines or innate immune parameters. Of interest from this study is that supplementation with BB-12^®^ or *L. casei* 431^®^ may be an effective means to improve immune function by augmenting systemic and mucosal immune responses to a challenge [69]. An additional study investigated the effect of the probiotic strain *L. casei* 431^®^ on immune responses to influenza vaccination and respiratory symptoms in healthy adults [73]. The study reported that the daily consumption of *L. casei* 431^®^ resulted in no observable effect on the components of the immune response to influenza vaccination, however it did record a reduction in the duration of upper respiratory symptoms.

## 4. Discussion

Although studies suggest that the administration of single and multiple species probiotic formulations to enhance vaccine immunogenicity is contentious, those studies that administered probiotic formulations containing *Bifidobacteria* species showed efficacy as adjuvants for vaccines in both children and adults more so than formulations with single species of *Lactobacilli* (Table 1 and Table 2). *Bifidobacteria* and *Lactobacilli* encourage the intestinal microbiota to exert a beneficial effect on mucosal immunity. It is hypothesised that there are probably a combination of both structural components (e.g., exopolysaccharides, bacteriocins, lipoteichoic acids and surface-associated and extracellular proteins) [75] and secreted factors (e.g., reactive oxygen species) belonging to these probiotic genera that enhance immunological responses to vaccinations. Recent studies in several animal models have significantly improved our understanding of the mechanisms by which the gut environment and intestinal microbiota affects the response to vaccines [41]. Studies have reported that intestinal resident macrophages are critical cellular components in the innate immune system response [76]. Consistent with their documented roles in maintaining immune equilibrium, in vitro studies with macrophages have reported that macrophages can establish an antiviral state reducing viral infections following contact with various species of *Lactobacilli* (e.g., *Lactobacillus paracasei*/*rhamnosus*) and *Bifidobacteria* (e.g., *Bifidobacterium longum*) [77]. This effect was reported to be probiotic species specific. In addition, probiotics have also been reported to modulate in vitro activation of T and natural killer cells. Live *Lactobacillus casei* Shirota strain that was co-cultured with primary human peripheral blood mononuclear cells in vitro showed an augmentation activation phenotypes and cytokine production [78]. Specifically, up-regulated expression of the activation markers CD69 and CD25 were observed on NK and CD8^+^ T cells, which are two important immune cell subsets with antiviral and vaccine-specific immune capacities. In addition, the Shirota strain augmented the production of IL-1, IL-6 and TNF-α, as well as the killing function of NK cells. Similar findings have been noted with other strains of both *Lactobacilli* and *Bifidobacteria* [78] suggesting that one potential mechanism of protection during infection is to promote antigen specific recognition and killing of infected monocytes in vivo, thereby limiting the spread of infection.

The use of probiotics as adjuvants for improving vaccine responses has significant plausible evidence, yet making firm conclusions regarding their adjuvant roles is still considered contentious. It is biologically plausible to continue to use probiotics until a vaccine schedule is completed, including throughout the duration of booster vaccinations. Adopting this approach the administration of probiotics could facilitate and enhance the longevity of vaccines. As for example, reported studies, which assessed the incidence and duration of cold and flu-like symptoms following influenza vaccinations, have reported results of decreased numbers of infectious incidents in those who also received probiotic supplementations [64,69].

Most of the studies investigating the impact of single- or multi-strain formulations of probiotics on responses to vaccinations have been conducted in healthy adults, and have only managed to report moderate improvements in immune responses [79]. A significant gap in the literature remains relevant to data from studies with unhealthy adults. Studies completed to date in healthy infant populations and the elderly are limited and therefore research finds it difficult to derive definitive conclusions and recommendations [79]. Yet, probiotic administration that encourages the intestinal microbiome to improve vaccine/immunotherapy efficacy remains a very plausible postulate.

The scientific evidence suggests that probiotics have the therapeutic potential to shorten the length of common respiratory conditions. A recent meta-analysis reported that, at least in adults administered vaccinations to protect against influenza viruses, the administration of probiotics and prebiotics was efficacious [80]. The effect is perhaps attributed to a fine–tuning of the mucosal barrier and metabolic system.

Data relating to the specific responses of specific vaccinations remains sparse, however, compelling data exists, which details how the intestinal bacteria influences the host immune responses, directly and indirectly, when exposed to viral and infectious diseases [79]. The current requisite therefore stands as further well designed, randomized, placebo-controlled studies to be conducted in order to clearly and fully understand the immune-modulatory properties of probiotics, whether the effects exerted are formulations or age-dependent and what their clinical relevance is in enhancing protection following vaccinations.

## 5. Conclusions

Studies that investigate the relationship between the intestinal microbiome and the development and function of the immune system continue to demonstrate novel concepts that increase knowledge based concepts for disease treatment. Cancer immunotherapy is such an example where for more than a decade in the field of oncology, the objective to harness the patient’s immune system to kill tumours has remained a key goal [81,82]. Recent research strongly suggests and shows that the intestinal.

immunotherapies [88] as well as an adjuvant for vaccines in early and late life. bacterial cohort can significantly facilitate the efficacy of checkpoint inhibitor immunotherapies in cancer treatments [83,84,85,86]. As a consequence of this research activity, the administration of probiotics (e.g., *Bifidobacterium breve*) as an adjuvant therapy for the modulation of chemotherapy efficacy and toxicity has been reported [87].

Equally the administration of vaccines from recent findings suggest complex mechanisms are in operation by which the microbiome impacts immune cell development and differentiation with the major implication being that the composition of the microbiome may ultimately affect vaccine efficacy. An intestinal resident immunity equilibrium is present that links the intestinal bacteria, the intestinal epithelia and the host’s immune response that leads to homeostasis maintenance. Resultant perturbations in this equilibrium with changes in the composition of the intestinal microbiome can result in chronic inflammatory processes (e.g., IBD) and autoimmune pathologies (e.g., allergy/asthma, diabetes). There is hence a logical step established for the inclusion and administration of probiotic formulations in the treatment of cancers with

## Figures and Tables

**Table 1:** Clinical studies investigating the effects of probiotics on vaccine responses in children.

Summary of Probiotic Adjuvant Effects to Vaccines			
Probiotic(s)	Method	Vaccine (Strain)	Biological Effect
*L. casei GG* (LGG)	5 × 10^10^ CFU b.i.d. at vaccination and for 1-week following	Oral rotavirus vaccine	Increase in rotavirus-specific IgM antibody secreting cells Significant rotavirus IgA and IgM seroconversion [49] Improved immunogenicity
*Streptococcus thermophilus* **(control)** vs. *S. thermophilus* and *L. casei* strain CRL431, *L. acidophilus* strain CRL730, oligofructose and inulin (test product)	Daily CFU doses: 95 × 10^8^ for 16-week for control 95 × 10^8^, 95 × 10^6^ and 95 × 10^6^ for respective test product strains	DTP-Hib/23-valent anti-pneumococcal vaccine	No difference between groups in antibody levels neither before nor after vaccination [51] Less hygienic environment reported Immunogenicity not improved
*L. rhamnosus* GG	1.8 × 10^10^ CFU q.d. from 36-week gestation until birth * maternal administration	DTaP, Hib, PCV7 vaccines	Decreased TT response in infants, decrease PCV response for some. Nil change in Hib/Treg [52] Immunogenicity not improved
*L. paracasei* ssp. *paracasei* strain F19	1 × 10^8^ CFU q.d. for 39-week	DTaP, polio and Hib vaccines	Probiotic enhanced anti-diptheria antibody titres in infants breastfed for less than six months [53] Early life effect (first 6-months) Improved immunogenicity
*L. acidophilus* LAVRIA1	3 × 10^9^ CFU q.d. for 26-week	Parenteral tetanus vaccine	Lower IL-10 responses to tetanus antigen in probiotic group [54] Early life effect (first 6-months) Improved immunogenicity
*B. breve* BBG-01	4 × 10^9^ CFU b.i.d. for 17-week	Oral cholera vaccine	Significantly lower responders and higher serum-LPS specific IgA in probiotic group and no difference in the vibriocidal antibodies [50] Similar immunogenicity responses, possible effect on intestinal microbiome
*L. acidophilus* ATCC4356 *B. bifidum* DSMZ20081 *B. longum* ATCC157078 *B. infantis* ATCC15697	3 × 10^9^ CFU q.d. for 20-week	MMRV vaccine	No difference in vaccine specific IgG antibody titres. Higher proportion reached protective IgG antibody titres in 3 month post-vaccination period in probiotic group [4] Improved immunogenicity
*B. longum* BL999 *L. rhamnosus* LPR	Total CFU q.d. 2.8 × 10^8^ for 26-week	Hep B vaccine at 1-month and DTPa/HepB vaccine at 6-months	Group treated with probiotics showed a trend towards increased antiHbsAg in infants given probiotic for six months [55] Early life effect (first 6-months) Improved immunogenicity
*L. rhamnosus* GG ATCC53103 *L. rhamnosus* LG705 *B. breve* Bbi99 *P. freundenreichii* ssp. *shermanii* JS	5 × 10^9^ CFU 5 × 10^9^ CFU 2 × 10^8^ CFU 2 × 10^9^ CFU 1 capsule b.i.d. to mothers for last gestation month and 1 capsule q.d. + 0.8 g galacto-oligosaccharides newborns for first 24-week	DTwP vaccine/Hib conjugate	Higher frequency of Hib-specific IgG antibody response and a trend for higher Hib-specific IgG GMT [56] Improved immunogenicity

^1^ DTP = diphtheria, pertussis and tetanus, Hib = *Haemophilus influenza* type b, DTaP = diphtheria, pertussis and tetanus, PCV7 = pneumococcal conjugate vaccine, MMRV = measles, mumps, rubella, varicella, HepB = Hepatitis B, DTPa = diphtheria, pertussis and tetanus, DTwP = diphtheria, pertussis and tetanus, *L. = Lactobacillus*; *B. = Bifidobacterium*; *P. = Propionibacterium*; ASCs = Antibody Secreting Cells; Ig = Immunoglobulin; CFU = Colony Forming Units, TT = tetanus toxoid; q.d. = once a day; b.i.d. = twice per day.

**Table 2:** Clinical studies investigating the effects of probiotics on vaccine responses in adults.

Summary of Probiotic Adjuvant Effects to Vaccines			
Probiotic(s)	Method	Vaccine (Strain)	Biological Effect
*S. thermophilus,* mesophilic streptococci*,* bifidobacteria Bb12*,* commercial mixed culture and *L. acidophilus* La1	1 × 10^7^–10^8^ CFU/g of both La1 and *Bifidobacteria* throughout study period (3-week)	*Salmonella typhi* Ty21a	Greater increase in vaccine-specific serum IgA antibody titre in probiotic vs. control group [60] Improved immunogenicity
*L. rhamnosus GG* OR *L. lactis* OR Placebo	4 × 10^10^ CFU q.d. for 1-week OR 3.4 × 10^10^ CFU q.d. for 1-week OR Ethyl cellulose for 1-week	Attenuated *Salmonella typhi* Ty21a oral vaccine	Greater increase in specific IgA in LGG group. *L. lactis* group had significantly higher expression of CR3 receptor [61] Improved immunogenicity
*L. paracasei* (NCC 2461)	1 × 10^9^ CFU + 6 g fructo-oligosaccharide daily for 52-week	Parental trivalent influenza vaccine	NK activity increased and less infections reported by supplemented group. Increased innate immunity and protection against infections in supplemented elderly [62] Improved immunogenicity (innate immunity)
*L. rhamnosus* GG OR *L. acidophilus* CRL431 OR Placebo	10^10^ CFU/serving q.d. for 5-week OR 100 g/day acidified milk without probiotics	Live attenuated poliomyelitis vaccine	Probiotic group reported increased poliovirus neutralizing antibody titers and poliovirus-specific serum IgA and IgG in probiotic group [63] Improved immunogenicity
*L. fermentum* CECT5716	1 × 10^10^ CFU containing capsule q.d. for 4-week	Inactivated trivalent influenza vaccine	Probiotic increased vaccine-specific IgA antibodies post-vaccination. Incidence of influenza-like illnesses for 5 months post-vaccination lower in the probiotic group [64] Improved immunogenicity
*B. lactis* (Bi-07 or B1-04) OR *L. acidophilus* (La-14 or NCFM) OR*L. plantarum* Lp-115 OR *L. paracasei* Lpc-37 OR *L. salivarius* Ls-33	1 × 10^10^ CFU/capsule b.i.d. for 3-week	Oral cholera vaccine	Significant changes in serum Ig concentrations in 6 out of 7 probiotic strains compared to control [65] Improved immunogenicity
*L. paracasei* subsp. *paracasei* DN-114 001	10^10^ CFU/bottle b.i.d. pilot study: 7-week confirmatory study:13-week	Parental trivalent influenza vaccine	Influenza-specific antibody titres increased in probiotic group post-vaccination with significantly greater seroconversion rate for B strain in confirmatory study [66] Improved immunogenicity
*L. rhamnosus* GG	1 × 10^10^ CFU + 295 mg Inulin b.i.d. for 4-week	Live-attentuated nasal influenza (LAIV)	Protection against H1N1 and B strain vaccine similar for placebo and probiotic group. H3N2 strain showed increased protective titer for LGG group [67] Improved immunogenicity
*L. casei* Shirota	1.3 × 10^10^ CFU q.d. for 176 days	Trivalent influenza vaccine	No statistically or clinically significant of *LcS* on protection against respiratory symptoms or improvement in seroprotection rates [68] No improved immunogenicity
*B. animalis* ssp. *lactis* BB-12 OR *L. paracasei* ssp. *paracasei* *L. casei* 431	1 × 10^9^ CFU q.d. for 6-week	Parental trivalent influenza vaccine	Significantly greater increase in vaccine-specific IgG antibody titre and mean-fold increases for vaccine-specific secretory IgA antibody in probiotic group [69] Improved immunogenicity
*Lactobacillus plantarum* CECT7315/7316	Group A: 5 × 10^9^ CFU q.d. for 12-week Group B: 5 × 10^8^ CFU q.d. for 12-week	Trivalent influenza vaccine	Consumption of probiotics for 3-mo following vaccination increased influenza-specific IgA and IgH antibody levels. Increasing trend in IgM antibodies also observed [70] Improved immunogenicity
Heat–killed *L. casei*	1 × 10^12^ CFU/daily dose for 12-week	Trivalent influenza vaccine	IgG, IgM, IgA did not change significantly in either group. HI titers against all 3 antigens significantly higher in probiotic group than baseline whereas only HI titers against A/H3N2 higher in placebo. Seroconversion rate against influenza antigens not statistically significant [71] No improved immunogenicity
*B. longum* BB536	5 × 10^10^ CFU/2 g sachet b.i.d. for 12-week, with 4-week additional follow-up	Trivalent influenza vaccine	Increase in IgA in probiotic group compared to placebo at wk 16. No significant effect on HI titers in probiotic group [72] Beneficial shift in intestinal microbiome Improved immunogenicity
*L. casei* 431^®^	10^9^ CFU q.d. 42 days	Trivalent influenza vaccine	Immune responses of probiotic group showed no effect but reported significantly shorter respiratory symptom durations (no differences for symptom incidence or severity) [73] No improved immunogenicity
*L. paracasei* MCC1849	10^9^ CFU q.d. for 6-week	Trivalent influenza vaccine	No significant differences in immune parameters between the groups. In oldest of the old subgroup (≥85 y.o) antibody responses to A/H1N1 and B antigens improved only in probiotic group. No significant effects of non-viable L. paracasei MCC1849 observed [74] Partial improved immunogenicity

^1^ *L*. = *Lactobacillus*; *B*. = *Bifidobacterium*; *P.* = *Propionibacterium*; ASCs = Antibody Secreting Cells; Ig = Immunoglobulin; CFU = Colony Forming Units; HI = Hemagglutination inhibition; y.o = years old; q.d. = once daily; b.i.d. = twice daily.
