# Correction: Vitetta, L.; et al. Adjuvant Probiotics and the Intestinal Microbiome: Enhancing Vaccines and Immunotherapy Outcomes. *Vaccines* 2017, *5*, 50

**DOI:** 10.3390/vaccines6020026

**Published:** 2018-05-15

**Authors:** Luis Vitetta, Emma Tali Saltzman, Michael Thomsen, Tessa Nikov, Sean Hall

**Affiliations:** 1 Sydney Medical School, The University of Sydney, Sydney 2006, Australia; esal4025@uni.sydney.edu.au (E.T.S.); mtho0952@uni.sydney.edu.au (M.T.); 2 Medlab Clinical Ltd., Sydney 2015, Australia; tessnikov@gmail.com (T.N.); sean_hall@medlab.co (S.H.)

The authors wish to make the following corrections to this paper due to a typesetting error in the conclusion of this article which was recently published in *Vaccines*. [1]. The conclusion in its entirety should read:

“Studies that investigate the relationship between the intestinal microbiome and the development and function of the immune system continue to demonstrate novel concepts that increase knowledge-based concepts for disease treatment. Cancer immunotherapy is such an example where for more than a decade in the field of oncology, the objective to harness the patient’s immune system to kill tumours has remained a key goal [81,82]. Recent research strongly suggests and shows that the intestinal bacterial cohort can significantly facilitate the efficacy of checkpoint inhibitor immunotherapies in cancer treatments [83–86]. As a consequence of this research activity, the administration of probiotics (e.g., *Bifidobacterium breve*) as an adjuvant therapy for the modulation of chemotherapy efficacy and toxicity has been reported [87].

Equally, the administration of vaccines from recent findings suggest complex mechanisms are in operation by which the microbiome impacts immune cell development and differentiation, with the major implication being that the composition of the microbiome may ultimately affect vaccine efficacy. An intestinal resident immunity equilibrium is present that links the intestinal bacteria, the intestinal epithelia, and the host’s immune response that leads to the maintenance of homeostasis. Resultant perturbations in this equilibrium with changes in the composition of the intestinal microbiome can result in chronic inflammatory processes (e.g., IBD) and autoimmune pathologies (e.g., allergy/asthma, diabetes). There is hence a logical step established for the inclusion and administration of probiotic formulations in the treatment of cancers with immunotherapies [88] as well as an adjuvant for vaccines in early and late life.”

The authors would like to apologize for any inconvenience caused to the readers by these changes.
